# Who Moves to Whom? Gender Differences in the Distance Moved to a Shared Residence

**DOI:** 10.1007/s10680-018-9490-4

**Published:** 2018-04-26

**Authors:** Maria Brandén, Karen Haandrikman

**Affiliations:** 1 Stockholm University Demography Unit, Department of Sociology, Stockholm University, 10691 Stockholm, Sweden; 2 The Institute for Analytical Sociology, Linköping University, 60147 Norrköping, Sweden; 3 Department of Human Geography, Stockholm University, 10691 Stockholm, Sweden; 4 Department of Social and Economic Geography, Uppsala University, Box 513, 75120 Uppsala, Sweden

**Keywords:** Union formation, Migration, Migration distance, Co-residence, Gender

## Abstract

Although the migration of couples and families is well examined, the migration that occurs at the start of co-residence has only been minimally studied. This study examines (1) whether women move more often and move over longer distances at the start of co-residence and (2) whether gender differences (if any) stem from compositional differences between women and men, such as gender differences in ties, or if they are the consequence of the within-couple distribution of bargaining power. The analyses are performed on Swedish population register data from 1991 to 2008, including longitudinal information on the residence of all couples who either married or had a child as cohabitants in 2008, backtracking them to the year of union formation. The results indicate that women are more prone to move for the sake of their male partner in the process of union formation than vice versa. If partners lived in close proximity prior to co-residence, the woman’s increased likelihood of moving and longer distance moved is nearly completely explained by power imbalances in the couple. Gender differences in ties only have minor importance in explaining gender differences in these types of migration patterns. If partners lived far apart prior to co-residence, gender differences are particularly pronounced. These differences remain after adjusting for the two partners’ relative resources. We contribute to the family migration literature by suggesting that women’s higher propensity to move and their longer distance moved are indications that even couples’ decisions at the start of co-residence are made in favour of the man’s career.

## Introduction

Family migration research has long acknowledged that couples’ migration decisions tend to be taken with the man’s career in mind rather than the woman’s (Cooke 2008a). Couples are more likely to move for the man’s work than for the woman’s work (Gordon 1995; Markham and Pleck 1986; Shihadeh 1991), and the man’s socio-economic, educational, and occupational characteristics often have a greater effect on a couple’s migration propensity than the woman’s characteristics [Duncan and Perucci 1976; Jacobsen and Levin 2000; McKinnish 2008; Nivalainen 2004; Shauman 2010; Shihadeh 1991; Swain and Garasky 2007; however, see Smits et al. (2003), for the Netherlands, and Brandén (2013a), for Sweden, for slightly contradicting results]. Men are also more likely to be employed following a move and to experience economic gains from moving (Cooke 2003; Cooke and Bailey 1996; Cooke et al. 2009; Shihadeh 1991; Smits 2001), whereas there is evidence of women experiencing economic loss from moving (Boyle et al. 1999; Brandén 2013b; Lichter 1983; Perales and Vidal 2013; Shauman and Noonan 2007).

A related but scarcely researched area focuses on the first move in a couple’s joint migration career, that is, the mobility which occurs when a couple starts their co-residential union. One recent study has examined the competing risks of living apart together (LAT)—couples to moving in together or to dissolve the union, finding that short-distance LAT couples have a higher likelihood to start co-residence than long-distance LAT couples (Krapf 2017), albeit it does not focus on the gendered aspect of such processes. Gender differences in the likelihood of moving and the distance moved when forming a co-residential union tend to have consequences for the partners’ future social and professional networks and careers, as well as for social ties (Løken et al. 2013). Research on family migration has shown that women’s careers suffer after long-distance moves (Boyle et al. 2003), and it is likely that migrating with the sole purpose of moving to a partner has similar consequences. Therefore, increased attention to this topic is warranted, particularly as there are indications that couples adapt to the man’s socio-economic and family situation in these types of moves. For example, in most countries, couples live in closer proximity to the man’s parents than the woman’s parents [Baker and Jacobsen (2007), Blaauboer et al. (2011), for the Netherlands; Malmberg and Pettersson (2007), for Sweden; Løken et al. (2013), for Norway; Huang (2012), for Taiwan]. Closer proximity to men’s parents, combined with an excess female migration propensity in the year of marriage and childbearing (Fischer and Malmberg 2001; Mulder and Wagner 1993), indicates that women are more likely to move and to move over longer distances at the start of co-residence than men. This is supported by a descriptive report from Statistics Sweden (2012). The report showed that, among all couples who had their first common child in the year 2000, 43% of all co-residential unions started with the woman moving in with the man, whereas men moved in with women in 32% of all cases.

Similar to other countries, most people who move within Sweden move over shorter distances. Usually, short-distance moves are associated with housing and family changes, while longer distance moves more often relate to employment. Based on survey data, Niedomysl (2011) reported that social reasons for moving, including union formation, are prominent across all moving distances in Sweden. Half of all partners who later married or had a child in the 1990s and 2000s, lived within 9 km of each other before moving in together (Haandrikman 2015). In the same study, distances moved were shortest in urban areas, for those with parents nearby, and for those born abroad. Migration over long distances, for instance to other labour market areas, is not that common: in 2001, 2.1% of all people moved between such areas (Lundholm 2007). Most long-distance movers are young adults, and long-distance movers tend to be more highly educated. In recent decades, student migration has gained greater importance at the expense of labour migration. Local ties, such as employment, marriage, and children, decrease migration propensities; the longer one has resided in an area, the more inclined the person is not to move (Fischer and Malmberg 2001; Lundholm 2007). Sweden is a leader in gender egalitarianism (Goldscheider et al. 2015), with women being as active in the labour market as men. However, migration decisions are still male-centred: women are more willing than men to move for their male partner’s career opportunities, and fathers are more likely to move for work than mothers (Brandén 2014).

To the best of our knowledge, the present study is the first to examine gender differences in migration propensities and distance moved at the start of co-residence. We use data from the Swedish high-quality, longitudinal population registers, containing detailed geographical attributes and partner characteristics before (and after) union formation. We study all couples who either experienced childbearing in a cohabiting relationship in 2008 or who married in 2008. Each partner is followed back in time until the last year they did not live at the same address. At this point in time, we measure the Euclidean distances between the addresses where the two partners lived when they were single and the address of their first joint home. We study whether women are more likely than men to move in with their partner, and whether female movers move over longer distances at the start of co-residence than their male counterparts. We also analyse whether these differences, if any, stem from (a) gender differences in characteristics that impact the likelihood and distance of moving, such as ties to the local, housing or labour market or (b) the relative bargaining advantages of the man in the couple, in terms of for instance income and labour market attachment.

## Mechanisms Underlying Gendered Moves at the Start of Co-residence

Moving at the start of co-residence can be understood as sitting between an individual’s and a couple’s moving—even though the move may be undertaken by either one or two individuals, it nevertheless results in a new joint household. In order to comprehend this process, a sensible point of departure lies in neoclassical economic theories on migration, which assume that individuals tend to take migration decisions in order to either maximise their own utility (Sjaastad 1962) or, alternatively, the couple’s pooled utility (Mincer 1978).

Each partners’ likelihood to move at the start of co-residence would, from this perspective, be determined by (1) the losses and (2) the gains the partner believe they could expect from moving. An individual’s losses from moving are intimately connected to the number and strength of ties the individual has to their current place of residence, that is, the strength of the links between an individual and a place. A large number of strong local ties tend to make migration less attractive, as it implies that there are advantages of the current place of residence that cannot be transferred to a new region (Fischer and Malmberg 2001). Increased age, being married, having school-aged children, having lived in a particular location for a long time, and having stable employment increase the strength of an individual’s ties to the current region of residence and, therefore, tend to make it more rewarding to stay than to move. These ties could also operate on a more local level, for example in terms of ties to one’s home or local community. Perceived gains from moving to a partner are closely connected to an individual’s past human capital investments, as well as the location specificity of such investments. Here, educational level is of large importance. For instance, the pay-off from moving, for a highly educated individual is, in general, high, and the move can even become an additional investment in one’s human capital if the future region of residence gives a higher pay-off from the education than the current region of residence (Sjaastad 1962). In the same way, an individual’s occupation may impact migration. As an illustration, working in a geographically flexible occupation (Halfacree 1995) often means having human capital that is easily transferrable between regions. On the other hand, being self-employed implies a more location-specific human capital, where a large proportion of one’s human capital, in terms of clients, knowledge of the local market, etc., is likely to be lost in the event of migration. This perspective posits that the partner with stronger local ties and less transferable human capital will have fewer incentives to move because the losses from moving will be greater.

For couple-level decisions, the two partners’ individual-level aspirations and wishes need to be weighed against each other for a decision to be made. Here, theories aimed at explaining family migration can guide us. Mincer (1978) argued that couples’ migration decisions are taken with the couple’s pooled utility in mind.^1^ This assumption has, however, been nuanced over many decades of family migration research (Cooke 2008a), and two main complementary theories have been developed to explain couple migration decisions, in particular, explaining why they tend to be favourable to the man rather than the woman. *The relative resource theory* (Blood and Wolfe 1960) builds on the notion that, in the event of a conflict of interests between the partners, the partner with the most (economic) resources will negotiate the most beneficial deal (Blood and Wolfe 1960; England and Kilbourne 1990; Lundberg and Pollak 1996, 2003). This means that the partner with the most power will drive the location decision, based on their own perceived benefits from moving, rather than on the couples’ pooled benefits. The theory assumes gender symmetry, that is, that a resource is as highly valued regardless of whether it belongs to the woman or the man. However, as the man’s economic resources often outweigh the woman’s (Magnusson 2010), the man will be likely to negotiate the more beneficial deal in family migration decisions, which, in turn, will often lead to family migration decisions that prioritise the man’s wishes over the woman’s (Lundberg and Pollak 2003). For instance, if the two partners live far apart prior to union formation, and the man has higher earnings than the woman, it is more likely that the woman moves to the man rather than vice versa. This mechanism is also applicable to short-distance moves. To illustrate, if the man has a housing advantage over the woman, where he owns his apartment whereas the woman doesn’t, it is likely that the couple start their co-residential union in the man’s residence rather than the woman’s. Furthermore, *traditional gender ideology* is a crucial predictor of how family migration decisions are taken (Bielby and Bielby 1992). Male breadwinner norms tend to make both women and men prioritise the man’s career at the expense of the woman’s. This often increases a couple’s likelihood of moving for the sake of the man’s career and decreases the likelihood of adapting to the woman’s career (Bielby and Bielby 1992; Brandén 2014; Cooke 2008b; Jürges 2006).

## Hypotheses

Building on the three empirical patterns linked to the theoretical discussion above, we expect women to move to the man more often, and to move over longer distances than the man, than vice versa.

First, there are compositional differences between populations of women and men regarding their local ties. The amount of local ties that women and men typically have at the start of co-residence generally differs in ways that make it more likely for the woman to move and to move over longer distances than the man. Women are typically younger than men when they start cohabiting (Kolk 2015; Presser 1975), which is associated with a weaker attachment to the labour market and lower housing quality (Mulder and Wagner 1993). Women are also, on average, enrolled in education for a longer period than men (Statistics Sweden 2012), which slows their labour market establishment and results in an unstable housing situation. Young men tend to move less often than young women [Lundholm (2007) for Sweden; Mulder and Wagner (1993) for West Germany], live in closer proximity to their parents than women do (Baker and Jacobsen 2007; Malmberg and Pettersson 2007; Løken et al. 2013; Chan and Ermisch 2015), and leave home later in many European countries (Chiuru and Del Boca 2010), increasing their local ties even more.

In addition, women’s human capital is more easily transferrable to new regions compared to men’s. For instance, women, on average, have a higher education than men (Buchmann et al. 2008; Statistics Sweden 2012), which is associated with being employed in occupations that exist all over a country (Brandén 2013a), and they are also overrepresented in occupations that are geographically flexible, such as in teaching and the care sector (Brandén 2013a; Shauman 2010). This occupational sex segregation partly explains why men’s careers seem to drive family migration decisions in the USA, the UK, and Sweden (Brandén 2013a; Perales and Vidal 2013; Shauman 2010; Shauman and Noonan 2007). All this may make women’s losses from moving lower than men’s, which would increase women’s likelihood to move at the start of co-residence.

Second, the man’s relative resources within a couple often outweigh the woman’s (Lundberg and Pollak 2003), both financially and in terms of age (Bozon 1991). Within-couple differences in socio-economic characteristics (Boyle et al. 1999) and age (Statistics Sweden 2012) have been linked to gender differences in family migration decisions and are likely to be important for moving to a first shared co-residence. Large age differences in favour of the man are associated with weaker bargaining power for the woman (Bozon 1991). Building on Lundberg and Pollak (2003), within-couple male advantage in relative resources, such as earnings, age, and housing characteristics, may lead to men moving less than women at the start of co-residence.

Third, the prevalence of traditional gender norms is likely to amplify women’s excess likelihood of moving at the start of co-residence and their distance moved, regardless of the distribution of measurable characteristics, such as those discussed above (Bielby and Bielby 1992). For example, gendered norms, even if only marginally in favour of the male breadwinner model over the dual-earner model, make it likely that couples in which partners are similar in terms of local ties, human capital, and relative resources will consider it to be a larger sacrifice for the man to give up his career at his current place of residence for moving than for the woman to do the same. This implies that gendered norms may make it more likely for the woman to move to the man than vice versa. This argumentation leads us to the following hypotheses:

### **Hypothesis 1**

Women are more likely to move and to move over longer distances at the start of co-residence than men.

### **Hypothesis 2**

Gender differences in the amount of local ties explain part of the gender differences in the likelihood of moving and in the distance moved.

### **Hypothesis 3**

Within-couple distribution of bargaining power explains part of the gender differences in the likelihood of moving and in the distance moved.

## Data and Methods

### Data

For the analyses, we used Swedish population register data. Our data include all individuals who were registered in Sweden during the period 1991–2008 and a wide range of demographic, socio-economic, housing, and migration variables. The database’s excellent geographical attributes, particularly location coordinates for each 100 m^2^ area, enable a longitudinal analysis of the distance moved for all inhabitants in Sweden. Since individual information can be linked over time, we can observe attributes prior to union formation.

In Swedish registers, individuals are registered at properties (*fastigheter* in Swedish) rather than in households. Couples are defined as two individuals who live in the same property and are either married to each other or have a common child (Thomson and Eriksson 2013). Individuals who cohabit without having a common child are, hence, not captured, as we do not know whether they live in the same or in different households within the same property. This drawback of the otherwise high-quality Swedish register data is discussed later in the paper. In the data, place of residence is approximated by the geographical coordinates of the 100 m^2^ area where the property is registered.

Couples included in our analysis include, first, those who were linked through a common child and the same property in 2008 but who had no common children in 2007. Second, we identified partners who married in 2008 (regardless of whether they had common children before marriage). For each couple, we then traced back year by year, until 1994, to examine where the partners lived the year before they first lived in the same 100 m^2^ area. We defined this time as the start of co-residence. We measured the Euclidean distance between the partners’ previous and new addresses in the year before partners lived in the same square. Due to the way partners are linked to each other in the data, first selecting couples and then backtracking their addresses in time is the only way to follow couples’ mobility over time. The final data set includes information on 139,722 individuals or 69,861 couples.

Some limitations are that, in the unlikely event of two individuals already living in the same square before they became a couple, the timing of co-residence was mistakenly measured prior to the start of actual co-residence. However, the determination of couples was not affected. We were unable to study partners who started cohabiting more than 15 years before they had children or married, as we had no information about them prior to union formation. In an attempt to examine whether our results are valid only for couples that co-resided for 15 years or less before marrying or having children, we examined whether migration patterns differed by the year that the partners started cohabiting, with no such indications. We would also like to highlight that the sample consists of ‘successful’ couples that stay together at least until they experience marriage or childbearing. Couples that dissolve before they marry or have joint children are hence not captured. If gender differences in moving to a partner are associated with the likelihood of ever having joint children or marrying, this may mean that our estimates will be biased to other populations than the one studied. However, even though this needs to be considered in the interpretation of our results, we know of no other data sets that are better suited for answering our research question. At the end of this paper, we discuss this issue in more depth. Finally, the quality of the data is dependent on whether people live where they are registered. Registered places of residence are derived from the population register at the Swedish Tax Agency and are regulated by Swedish law (Folkbokföringslagen 1991: 481, paragraph 6). The registers are considered to be of high quality (the Swedish Tax Agency 2006). For two groups, recent home leavers (Statistics Sweden 2008) and students (the Swedish Tax Agency 2006), the quality is somewhat poorer. To ensure that this lower-quality data did not bias our results, we repeated the analyses excluding individuals who were living with parents or studying the year before co-residence, with virtually identical results.

### Analytical Strategy

We examined the following two outcomes: (1) the likelihood of moving, i.e. contrasting a move to the partner’s residence or a new joint residence to only one partner moving and (2) the natural logarithm of the distance moved at the start of co-residence among those who move. The likelihood of moving was examined using logistic regression (and linear probability models in sensitivity checks), whereas gender differences in the distance moved (among movers) were studied using ordinary least squares (OLS) regressions. The analyses are performed at the individual level, in order to derive a gender coefficient that enables us to draw conclusions on whether women (1) are more likely to move at the time of union formation and (2) move over longer distances at the time of union formation, as well as (3) to examine how a number of proposed mechanisms can explain any such patterns. The standard errors are adjusted because the observations of the man and the woman in the couple are not independent of one another, using Stata’s cluster command.

We repeat all analyses including only couples where partners lived more than 50 km apart prior to union formation. If partners lived this far apart prior to union formation, at least one of the partners must have moved a considerable distance for the couple to start their co-residential union. Therefore, these individuals are theoretically interesting to examine separately, especially because of the impact that long-distance migration may have on location-specific networks and social ties (Løken et al. 2013). Furthermore, long-distance moves have been shown to have particularly detrimental effects on women’s labour market outcomes (see, e.g. Boyle et al. 2003). The 50-km threshold is commonly used to define a long distance in migration studies in Sweden (see, for example Malmberg and Pettersson 2007).

### Independent Variables

All characteristics of individuals and their (future) partners were measured the year prior to co-residence. At the individual level, we incorporated the following potential mediators to capture gender differences in local ties. Age and squared age were included as continuous variables. To adjust for young women’s generally higher mobility, we included a variable that indicated whether an individual changed county of residence during the three years preceding the start of co-residence (Sweden consists of 21 counties) and a variable that measured whether the individual had ever lived in his or her partner’s region of residence prior to co-residence.^2^ For the same reason, we included a categorical variable that measured the Euclidian distance between an individual and their closest living parent, as well as a binary variable on living in one’s parental home before co-residence. We further controlled for whether an individual had any underage children registered in their household.^3^

To adjust for gender differences in labour market attachment, we included a dichotomous variable on whether an individual was employed in November in all 3 years prior to living together, and a variable that indicated whether an individual was enrolled in education the year prior to co-residence (measured as whether an individual received study grants or loans). We captured gender differences in commuting patterns using a variable that distinguished between (1) living and working in the same place, (2) living and working in different places but not working in the partner’s place of residence, and (3) living and working in different places with the place of work the same as the partner’s place of residence, with places operationalised as a municipality. Educational level was measured as the highest educational level achieved in June. To account for geographically flexible jobs, we included a dummy variable for whether the individual works in the education, health, or social work sector, as previous studies have shown that individuals working in these highly female-dominated sectors tend to be more inclined to move (Brandén 2013a).

Income was measured as annual disposable income, deflated to values as of 2008, with values categorised in quartiles. We also included a variable that measured whether the individual lived in an owner-occupied dwelling before co-residence. This information was derived from the property register, in which properties are categorised based on owner form and type. Individuals were linked to the property registers of 1991, 1996, 2001, and 2006 (taking the most recent year prior to co-residence) using a unique property identification key. As the register is at the property level rather than the individual level, we only know whether individuals live in a property that is dominated by a certain tenure form. People who sublet an apartment in a house dominated by owner-occupied apartment housing are listed as owners. In order to adjust for regional variation in poverty, housing prices, employment opportunities, women’s overrepresentation in the urban areas of Sweden (Glesbygdsverket 2008), and young individuals’ preference to live in urban areas (Stockdale and Catney 2014), we included county of residence. We adjusted for neighbourhood affluence by incorporating the median disposable income of the population in the individual’s SAMS area (Small Areas for Market Statistics; Sweden consists of approximately 9000 SAMS areas that are rather homogeneous in terms of housing tenure, and SAMS are most commonly used geographical units in studies on residential mobility in Sweden) prior to co-residence. We transformed the variable to rank percentiles and included it as a continuous variable.

The individual’s relative resources with respect to their partner were operationalised by a set of relational variables in terms of the socio-economic characteristics and labour market attachment of the individual compared to the partner, measured in the year before co-residence (Boyle et al. 1999; Shauman 2010). All variables (except the distribution of income) distinguish between (1) same levels, (2) individual advantage, and (3) partner advantage. One of the partners is considered as having an *age advantage* if they are at least 3 years older than the other partner. *Income advantage* is measured by how much the individual and their partner would have contributed to the total couple income if the couple pooled their income in the year before co-residence (Sørensen and McLanahan 1987). This measure was calculated as (IncEGO/IncEGO + IncPARTNER)-(IncPARTNER/IncEGO + IncPARTNER) and varied between − 1 (the partner contributes all of the total couple income) and + 1 (the individual contributes all of the total couple income).^4^*Area advantage* is operationalised by first calculating the zero-centred difference in neighbourhood affluence of the two partners. (Neighbourhood affluence is operationalised as above.) If the zero-centred difference is larger than half a standard deviation of the mean (zero), the partner in the higher income area is considered as having an area income advantage (Shauman 2010). *Educational advantage* is measured as whether either of the partners has a higher educational level than the other, *housing advantage* is measured as whether one of the partners owns their home while the other does not, and *employment advantage* is measured as whether one of the partners has had stable employment during the previous 3 years while the other did not.

## Descriptive Results

Table 1 shows descriptive statistics for moving patterns at the start of co-residence in Sweden. It was more common for the woman to move to the man at the start of co-residence than vice versa. Interestingly, more than half of all co-residential unions started with both partners moving to their first joint residence. Among movers, women generally moved over longer distances than men. On average, female movers moved 59 km when forming a union, whereas male movers moved 50 km. This pattern was amplified when partners lived far apart prior to co-residence. If the two partners lived in close proximity, there were no gender differences in the mean distance moved to a first shared residence. Similar patterns were reflected in the median distance that women and men moved.

**Table 1:** Moving patterns at the start of co-residence in Sweden. *Source*: Swedish register data, authors’ calculations

		Men	Women
Move or not (percentages)	Stay: partner moves in	28	21
	Move: to partner	21	28
	Move: to new joint home	52	52
Distance moved (mean in kilometres)	All movers	50	59
	Movers and partners lived < 50 km apart	19	19
	Movers and partners lived > 50 km apart	150	183
Distance moved (median in kilometres)	All movers	6	8
	Movers and partners lived < 50 km apart	4	5
	Movers and partners lived > 50 km apart	76	114
*N*	All	69,861	69,861
	Movers	50,445	55,408
	Movers and partners lived < 50 km apart	38,685	41,969
	Movers and partners lived > 50 km apart	11,760	13,439

Table 2 includes descriptive statistics for the potential mediators of gender differences in migration patterns. All variables were measured the year prior to living together, unless otherwise specified. We found clear gender differences in most individual characteristics prior to co-residence. Some highlights are that women were generally younger than men and, hence, moved straight from the parental home to co-residence more often. Men were more strongly attached to the labour market than women, while women were in education more often. Men commuted to a larger extent than women. One-third of all women worked in the education, health, or social work sector the year before the start of co-residence, whereas only 9% of men worked in these sectors. Men were more likely than women to be owner-occupiers. The relational variables painted roughly the same picture as the individual-level variables, i.e. within couples, the man often had a relative bargaining advantage compared to the woman. Table 2 also shows that partners who lived more than 50 km apart before co-residence had fewer ties to their current home region, compared to all couples. Long-distance couples were more geographically mobile during the years preceding co-residence, had children less often, lived farther away from both sets of parents, had been continuously employed less often, and commuted or were in education the year prior to co-residence more often.

**Table 2:** Descriptive statistics of main independent variables. *Source*: Swedish register data, authors’ calculations

	All new couples		Partners lived > 50 km apart	
	Men	Women	Men	Women
Age (mean)	31	29	31	29
Changed county in last 3 years (%)	14	17	24	28
Has previously lived in partner’s county of residence (%)	83	84	34	36
Distance to closest parent (%)				
< 5 km	48	47	44	43
5–50 km	23	22	19	17
50 km or longer	19	22	27	32
Parents deceased or not living in Sweden	10	9	9	9
Lives in the parental home (%)	22	24	24	26
Children in household (%)	6	20	5	15
Continuously employed the 3 years before co-residence (%)	61	47	56	42
In education (%)	17	35	23	41
Commuting (%)				
Did not commute	67	71	61	63
Commuted to other municipality	27	22	31	28
Commuted to municipality where future partner lived	6	6	8	9
Education level (%)				
Primary school	13	15	11	12
Upper secondary school	55	47	49	42
Tertiary education < 2 years	9	9	11	11
Tertiary education 2 years +	22	28	27	33
Missing	1	1	1	1
Work in care, teaching or social work sector (%)	9	32	10	32
Income (%)				
1st quartile	20	30	23	33
2nd quartile	22	29	22	29
3rd quartile	26	24	24	22
4th quartile	33	17	32	16
Housing (%)				
Rent	38	41	37	41
Owned housing	55	52	55	52
Missing housing	7	7	8	7
Area’s median income percentile (mean)	49	48	46	45
Age advantage (%)				
Same	45	45	47	47
Ego	44	11	43	10
Partner	11	44	10	43
Income advantage (mean)	0.11	− 0.11	0.11	− 0.11
Area advantage (%)				
Same	45	45	32	32
Ego	28	27	34	34
Partner	27	28	34	34
Educational advantage (%)				
Same	51	51	49	49
Ego	22	25	22	27
Partner	25	22	27	22
Either missing	2	2	2	2
Housing advantage (%)				
Same	50	50	48	48
Ego	20	17	21	17
Partner	17	20	17	21
Either missing	13	13	14	14
Employment advantage (%)				
Same	63	63	62	62
Ego	25	12	26	12
Partner	12	25	12	26
*N*	69,861	69,861	16,228	16,228

## Results of Multivariate Analyses

The modelling strategy consists of the following steps (Tables 3, 4, 5): with Model 1, we test Hypothesis 1, only including gender; Model 2 adds individual-level control variables to test Hypothesis 2; and Model 3 includes relational variables using both the individual and partner’s characteristics to measure men’s relative bargaining advantage, following the work of Shauman (2010) and Boyle and colleagues (1999), thereby testing Hypothesis 3.

**Table 3:** Logistic regression on the likelihood of moving when forming a union in Sweden. Odds ratios. *Source*: Swedish register data, authors’ calculations

	Model 1		Model 2^a^		Model 3^b^	
	Odds ratio	*p* value	Odds ratio	*p* value	Odds ratio	*p* value
Woman compared to man	1.48	0.000	1.44	0.000	1.08	0.000
Age			0.99	0.083	0.98	0.000
Age squared			1.0000^c^	0.909	1.0001	0.010
Changed county in last 3 years			1.26	0.000	1.26	0.000
Has previously lived in partner’s county of residence			1.03	0.042	1.01	0.533
Distance to closest parent (compared to < 5 km)						
5–50 km			1.11	0.000	1.10	0.000
50 km or longer			1.35	0.000	1.36	0.000
Parents deceased or not living in Sweden			1.11	0.000	1.16	0.000
Lives in the parental home			5.84	0.000	5.84	0.000
Children in household			0.65	0.000	0.67	0.000
Continuously employed the 3 years before co-residence			0.95	0.003	1.11	0.000
In education			1.10	0.000	1.12	0.000
Commuting (compared to did not commute)						
Commuted to other municipality			1.07	0.000	1.08	0.000
Commuted to municipality where future partner lived			1.47	0.000	1.45	0.000
Education level (compared to primary school)						
Upper secondary school			1.00	0.939	0.97	0.224
Tertiary education < 2 years			0.97	0.335	0.95	0.076
Tertiary education 2 years +			1.04	0.094	0.99	0.675
Missing			0.92	0.182	0.89	0.272
Work in care, teaching or social work sector			1.08	0.000	1.08	0.000
Income (compared to 1st quartile)						
2nd quartile			0.87	0.000	1.04	0.075
3rd quartile			0.76	0.000	0.96	0.155
4th quartile			0.73	0.000	0.97	0.292
Housing (compared to rent)						
Owned housing			0.81	0.000	0.87	0.000
Missing housing			0.96	0.103	1.04	0.361
Area’s median income (percentile)			1.00	0.000	1.00	0.001
Age advantage (compared to same)						
Ego					0.79	0.000
Partner					1.22	0.000
Income advantage					0.53	0.000
Area advantage (compared to same)						
Ego					0.88	0.000
Partner					1.16	0.000
Educational advantage (compared to same)						
Ego					0.97	0.069
Partner					0.99	0.558
Either missing					0.97	0.626
Housing advantage (compared to same)						
Ego					0.85	0.000
Partner					1.07	0.002
Either missing					0.93	0.012
Employment advantage (compared to same)						
Ego					0.79	0.000
Partner					1.18	0.000
Constant	2.60	0.000	3.30	0.000	3.56	0.000
*N*	139,722		139,722		139,722	
LL	− 76,901		− 70,723		− 69,433	
Pseudo *R* square	0.01		0.09		0.10	

^a,b^Models 2 and 3 include dummies for county of residence

^c^1.000005

**Table 4:** OLS regressions of the log distance moved at start of co-residence in Sweden. Unstandardised coefficients. *Source*: Swedish register data, authors’ calculations

	Model 1		Model 2^a^		Model 3^b^	
	*B*	*p* value	*B*	*p* value	*B*	*p* value
Woman compared to man	0.22	0.000	0.23	0.000	0.15	0.000
Age			0.04	0.000	0.05	0.000
Age squared			− 0.0003	0.000	− 0.0004	0.000
Changed county in last 3 years			0.42	0.000	0.41	0.000
Has previously lived in partner’s county of residence			− 2.07	0.000	− 2.02	0.000
Distance to closest parent (compared to < 5 km)						
5–50 km			0.27	0.000	0.26	0.000
50 km or longer			0.52	0.000	0.52	0.000
Parents deceased or not living in Sweden			0.18	0.000	0.20	0.000
Lives in the parental home			0.75	0.000	0.74	0.000
Children in household			− 0.16	0.000	− 0.16	0.000
Continuously employed the 3 years before co-residence			− 0.09	0.000	− 0.09	0.000
In education			0.11	0.000	0.10	0.000
Commuting (compared to did not commute)						
Commuted to other municipality			0.28	0.000	0.28	0.000
Commuted to municipality where future partner lived			1.19	0.000	1.16	0.000
Education level (compared to primary school)						
Upper secondary school			− 0.03	0.116	− 0.04	0.038
Tertiary education < 2 years			− 0.10	0.000	− 0.12	0.000
Tertiary education 2 years +			− 0.03	0.171	− 0.06	0.015
Missing			− 0.19	0.001	− 0.07	0.390
Work in care, teaching or social work sector			− 0.03	0.034	− 0.03	0.026
Income (compared to 1st quartile)						
2nd quartile			− 0.13	0.000	− 0.10	0.000
3rd quartile			− 0.18	0.000	− 0.15	0.000
4th quartile			− 0.20	0.000	− 0.16	0.000
Housing (compared to rent)						
Owned housing			0.12	0.000	0.23	0.000
Missing housing			0.08	0.002	0.07	0.034
Area’s median income (percentile)			0.00	0.218	0.00	0.000
Age advantage (compared to same)						
Ego					− 0.15	0.000
Partner					0.04	0.008
Income advantage					− 0.11	0.000
Area advantage (compared to same)						
Ego					0.32	0.000
Partner					0.47	0.000
Educational advantage (compared to same)						
Ego					0.01	0.443
Partner					0.00	0.909
Either missing					− 0.13	0.052
Housing advantage (compared to same)						
Ego					− 0.05	0.001
Partner					0.19	0.000
Either missing					0.10	0.000
Employment advantage (compared to same)						
Ego					− 0.04	0.021
Partner					− 0.01	0.478
Constant	8.81	0.000	9.04	0.000	8.58	0.000
*N*	105,853		105,853		105,853	
*R* square	0.00		0.21		0.22	

^a,b^Models 2 and 3 include dummies for county of residence

**Table 5:** Effect of gender on the likelihood of moving and the distance moved in Sweden for subgroups, women compared to men (ref.). Unstandardised coefficients. *Source*: Swedish register data, authors’ calculations

	A: Linear probability models of likelihood of moving (Coeff.)			B: OLS on log(metres moved) (Coeff.)	
	All	Partners lived < 50 km apart	Partners lived > 50 km apart	Partners lived < 50 km apart	Partners lived > 50 km apart
Model 1	0.07***	0.06***	0.10***	0.08***	0.60***
Model 2	0.06***	0.05***	0.08***	0.08***	0.67***
Model 3	0.01**	0.00	0.04***	0.05***	0.43***

**p < .01; ***p < .001

Table 3 shows the odds ratios from the logistic regressions of the likelihood of moving at the start of co-residence. Comparisons of the size of coefficients across models can be problematic in logistic regressions (Mood 2010). Hence, we repeated the analyses using linear probability models, that is, a linear ordinary least square (OLS) regression with the dichotomous outcome, (0) do not move and (1) move [as suggested by Mood (2010)], and robust standard errors. These complementary analyses are presented in Table 5.

The results indicate that women were more likely to move when forming a union than men (Table 1, Model 1), and from Table 5, Model 1, we learn that women were 7 percentage points more likely to move, compared to men (in line with the descriptive results in Table 1). This gender effect remained significant and largely unchanged even after adjusting for gender differences in the amount of local ties (Model 2).

The impacts of the individual-level variables were mostly in line with expectations (Table 3, Model 2). We found higher migration propensities among individuals with few local ties, such as those who had changed county of residence during the last 3 years or who lived far from their parents. Individuals with children had a lower likelihood of moving. The more labour market ties an individual had, the less likely the person was to move, as illustrated by the finding that individuals who did not have stable employment, were in education, or commuted before co-residence were more likely to move (Model 2). Interestingly, educational achievement did not have a significant impact on the likelihood of moving at the start of co-residence. Note that we adjusted for the generally higher migration propensity of highly educated individuals, by controlling for their mobility the 3 years prior to co-residence. Working in the care sector or in teaching was associated with an elevated likelihood of moving, whereas increased income was associated with a lower likelihood of moving at the start of co-residence. Living in owned property was associated with a decreased likelihood of moving.

Finally, in Model 3, we included indicators for the relative bargaining situation of the two partners. Again, the overall results were in line with our hypotheses. The odds ratio for gender was reduced from 1.48 to 1.44 between Models 1 and 2, and from 1.44 to 1.08 between Models 2 and 3 (which corresponds to a decrease in probabilities from 0.07 to 0.06 between Models 1 and 2, and a decrease from 0.06 to 0.01 between Models 2 and 3, see Table 5). Aside from that, there are few differences in the coefficients comparing Model 3 to Model 2.^5^ If the partner was older it increased the likelihood of moving, even when adjusting for housing situation and within-couple differences in housing situation. The more the individual earned, compared to their partner, the lower their likelihood of moving. When relative income was added to the model, individual-level income no longer had an impact on the likelihood of moving, indicating that relative income, rather than individual income, is crucial. The likelihood of moving increased if the partner lived in a more affluent neighbourhood than the individual, if the partner lived in an owned dwelling but the individual did not, or if the partner was continuously employed but the individual was not.

Table 4 shows the unstandardised coefficients of the OLS regressions of log distance moved when forming a union.^6^ Only individuals who moved were included in these analyses. The results of Model 1 show that, among individuals who moved, women moved over longer distances than men. Based on the coefficients from Table 4, we calculated women’s excess migration distance using the formula 100 × exp(0.22) = 125. This indicates that female movers, on average, moved 25% further than male movers. The coefficient is almost identical in Model 2. In Model 3, including both individual and relational variables, women’s excess distance moved was reduced to 16% (100 × exp(0.15) = 116).

Most of the results in Table 4 are in line with those found in Table 3. One difference compared to Table 3 is that, among movers, home ownership is not found to reduce the distance moved. In addition, among movers, those who had ever lived in the partner’s county of residence, on average, moved a shorter distance than those who had not.^7^ In contrast to the analyses on the likelihood of moving, working in female-dominated occupations reduced the distance moved, given that an individual moved.

Table 4, Model 3 shows that, among movers, being the older partner was associated with a shorter distance moved and economic bargaining power does impact the distance moved. Individuals whose partner lived in a more affluent area moved over longer distances than if partners lived in similarly affluent areas. However, this was also the case if the individual lived in a more affluent area. Even after adjusting for all of these factors, a 16% additional migration distance for women remained unexplained.

Table 5 shows the gender coefficients of a modified set of analyses with similar structure as those presented in Tables 3 and 4, but with slightly different model specifications. The analyses presented in column A are equivalent to Table 3; however, rather than logistic regressions, column A includes linear probability models of the likelihood of moving (as previously discussed). This makes it possible to compare coefficients across models. We performed separate analyses for couples for which the partners lived close (< 50 km) and far apart (> 50 km) prior to co-residence. Column B includes similar analyses as those presented in Table 4 but with separate analyses according to whether partners lived close or far apart prior to co-residence.

The effect of gender on the likelihood of moving when forming a union was reduced over all models, as shown in the results in column A. For couples in which the partners lived in close proximity prior to co-residence, no excess female migration probabilities remained in Model 3. If partners lived far apart prior to co-residence, women were 4 percentage points more likely than men to move, even after adjusting for all of the proposed mediators (column A, “Partners lived > 50 km apart”, Model 3).

Column B shows that, among movers, the natural logarithm of metres moved remained significantly higher for women across all models. If partners lived far apart prior to co-residence, on average, women moved 54% further than did men (100 × exp(0.43)), after adjusting for all of the proposed mediators. If partners lived in close proximity before co-residence, only 5% of the excess distance remains in Model 3. Hence, if partners lived in close proximity prior to co-residence, there were no substantial gender differences in the distance moved after adjusting for men’s relative bargaining advantage.

## Discussion and Concluding Remarks

To shed light on migration at the start of co-residence, we utilised the strength of a number of Swedish population registers to examine whether women move more often and over longer distances at the start of co-residence (Hypothesis 1), as well as whether gender differences (if any) are the consequence of compositional differences between women and men, such as gender differences in local ties (Hypothesis 2) and/or relative resources (Hypothesis 3).

Our findings show that women are more likely to move and, on average, move over longer distances at the start of co-residence than men, confirming Hypothesis 1. This is particularly pronounced for those living far apart before cohabitation. Our second hypothesis predicted that gender differences in the likelihood to move and the distance moved could be explained by overall gender differences in the amount of local ties, such as labour market attachment, housing situation and previous mobility patterns. This was tested by how the gender coefficient changed between a model including gender only (Model 1), and a model including gender and a number of indicators of local ties (Model 2). The similarity of the gender coefficients in the two models indicates that even though local ties explain part of the overall likelihood to move and the distance moved at union formation, they do not add any substantial value in explaining why women are more likely to move and generally move over longer distances than men, thus rejecting Hypothesis 2.

More importantly in explaining women’s higher likelihood to move and their longer migration distances was their relative bargaining disadvantage as compared to their male partner, confirming Hypothesis 3. For those living close to each other before cohabitation, nearly all gender differences in the likelihood to move and the distance moved stem from the man’s relative bargaining advantage in terms of age, employment, income, housing, and neighbourhood compared to that of the woman. One key mechanism behind why the woman seems to adapt more to the man than vice versa is, hence, that the man has an advantageous bargaining position, for example in terms of being more established on the labour market, being older, and having a better housing situation.

Long-distance migration is likely to have far greater consequences than residential mobility, which is why we repeated our analyses including only couples where at least one partner had to move a substantial distance to start the co-residential union. Our findings show that the relative bargaining advantage is also important if partners lived far apart prior to co-residence. However, for these couples, a substantial share of the gender differences remains unexplained, even after adjusting for this. The remaining excess female likelihood to move and the excess distance moved might be interpreted as indicators of traditional gender norms making the woman adapt more to the man than vice versa, although this cannot be explicitly tested with the current data set.

Women’s generally higher propensity to move and their long-distance moves are indications that couples’ decisions about mobility at the start of co-residence are made in favour of the man’s career, as is commonly found in the literature on family migration (Cooke 2008a). Women systematically adapt more to their partner’s career when couples decide where to live, and this is reflected in our findings. Furthermore, Sweden has a tradition of patrilocal marriage patterns (Pettersson and Malmberg 2009), which probably also drives the present results. An alternative explanation for the gender differences is that women are more often the initiators of co-residence, if we assume that moving in with the man or moving over longer distances indicates this is initiated by the woman. It has been suggested that women may drive family dynamics more than men (Andersson et al. 2006). For instance, women initiate divorce more often than men (Brinig and Allen 2000; Hewitt et al. 2006), and lesbian marriages have a higher union dissolution risk than marriages of gay men (Andersson et al. 2006). In line with women’s higher propensity to end a relationship, women may also be more keen on initiating co-residence; thus, they may be more likely to move to their partner than vice versa.

This study focused on couples that had a first common child or were married in 2008, and all other couples were excluded. This touches upon the problem with anticipatory analysis (Hoem and Kreyenfeld 2006), as we exclude couples who dissolved before marrying or having children, those that never marry or have children, as well as those that cohabited for more than 15 years before any of these events occurred. Family migration is associated with an increased risk of union dissolution (Boyle et al. 2008), and LAT couples that live far apart are more likely to dissolve than LAT couples that live within an hour from another (Krapf 2017). Our study population may therefore underestimate the number of couples that live far from another prior to co-residence. Another related issue is that if couples are less likely to marry or have children depending on whether the woman moves to the man than vice versa, our gender coefficient may be biased. For example, given that the woman more often moves to the man than the other way around, if couples where the woman moves to the man are more likely to dissolve before marrying of having joint children, we may underestimate the gender coefficient. We can be certain that our results are valid for couples that ever marry or have joint children, but some caution is therefore advised for inference across other populations. Even with these drawbacks, we know of no better suited data to answer our research question. However, Statistics Sweden is currently working on the possibility of providing researchers with data that can link unmarried cohabiters based on their address, which will greatly improve future analyses.

One important remaining issue is whether similar results can be found in other contexts or if these results are specific for Sweden. Although gender equality is widely embraced in Sweden, the commonly found patterns of men driving, and gaining from, family migration decisions are evident in Sweden (Åström and Westerlund 2009; Axelsson and Westerlund 1998; Brandén 2013c; Lundholm 2007; Nilsson 2001). Similar results might, therefore, be found in other contexts. However, the results may be stronger in magnitude and more stable after adjusting for differences in bargaining power in countries with less egalitarian gender norms. We encourage future research that addresses similar questions in other contexts.

The topic of migration at the start of co-residence remains underexplored. The impact that these moves have on the subsequent life trajectories of the moving partner(s) is unknown. It is possible that some women become disadvantaged from moving these long distances, for example by losing place-specific networks and career opportunities. This notion is in line with propositions in the family migration literature. However, it is also possible that some women move to a region that benefits them and adapt easily to the new region of residence, perhaps due to their skills for creating new social networks. Some researchers have argued that couples take turns in migration decisions, such that the tied mover determines the destination of the next move (Pixley 2008). Ideally, migration should be analysed as multiple decisions over the life course rather than a single residential move. Furthermore, an examination of the association between the risk of union dissolution, as well as the woman’s economic dependence, and inequality in migration distance at the start of co-residence should be conducted to add depth to our findings.

## Footnotes

1 Mincer (1978) argued that women became tied movers because the man’s individual-level utility from moving makes the couple-level utility from moving positive, even when the woman in the couple is not likely to benefit individually from it because of her weaker labour market participation and lower earnings.

2 Since 1990.

3 Most children of separated or divorced parents are registered with their mother in Sweden (Lundström 2009), even though many, in practice, share residence with both parents. Thus, the current measure underestimates the number of single fathers before co-residence. We, therefore, repeated the analyses without the variable of children in the household, with identical results.

4 We have also elaborated on other measures, such as first calculating the zero-centred difference in income between the two partners. If the zero-centred difference in income is larger than half a standard deviation of the mean (zero), the higher earning partner is considered as having an income advantage (Shauman 2010). Results are similar using this alternative measure.

5 Age and age squared become significant, while individual income and having lived in partner’s county of residence become insignificant; however, their respective effect sizes remain virtually identical. The effect of having stable employment changes from having a small negative effect to having a small positive effect on the likelihood of moving.

6 As the distance distribution among those living close to each other before co-residence is relatively skewed, we use the log distance instead.

7 Individuals who lived in their partner’s county of residence prior to cohabitation were categorised as having always lived in the partner’s county of residence. Consequently, if the partners lived in the same county prior to co-residence, the distance that either of the partners had to move was shorter.
